# The oral health and periodontal diseases awareness and knowledge in the Iraqi population: Online‐based survey

**DOI:** 10.1002/cre2.304

**Published:** 2020-06-26

**Authors:** Hayder R. Abdulbaqi, Ali A. Abdulkareem, Muhanad L. Alshami, Mike R. Milward

**Affiliations:** ^1^ College of Dentistry University of Baghdad Baghdad Iraq; ^2^ Department of Dentistry Dijlah University College Baghdad Iraq; ^3^ College of Medical and Dental Sciences University of Birmingham Birmingham UK

**Keywords:** dental health, oral health, periodontal diseases

## Abstract

**Objectives:**

This study aimed to evaluate oral health (OH) and periodontal diseases (PD) awareness in the Iraqi population.

**Material and methods:**

This study was a questionnaire‐based online survey of two weeks duration. The questionnaire was built using a Google platform and was distributed randomly via social media (Facebook and Telegram). The questionnaire consisted of a demographic data section and two other main sections for the evaluation of OH and PD awareness. Each response was marked with “1” for a positive answer and “0” for the other answers. For each respondent, answers were summed to give an overall score. The frequency of positive responses was used to determine the association of awareness with demographic data and the level of awareness into low, moderate, and high levels.

**Results:**

A total of 1,465 were included in the final analysis after application of exclusion criteria. The respondents showed significantly higher levels of awareness about PD (mean ± SD = 3.66 ± 1.42) than OH awareness (mean ± SD = 2.19 ± 1.29). Analysis of data showed that OH awareness was mainly associated with high degree holders (OR 1.851) and age > 45 years (OR 1.730). However, PD awareness did not show any evident association with demographic variables investigated. In general, the respondents exhibited low levels of OH knowledge and low to moderate level of PD knowledge.

**Conclusions:**

Despite limitations, this study revealed inappropriate levels of OH and PD awareness and knowledge in the Iraqi population and provided the baseline data necessary for the development of Governmental educational programs and health awareness campaigns which are highly suggested particularly focusing on the primary and high schools, in an attempt to improve the levels of awareness.

## 1 INTRODUCTION

The use of appropriate oral hygiene measures is a key to maintaining a healthy dentition and periodontal tissues. In addition, adopting good oral hygiene regimes has a positive impact on the individual's general wellbeing and quality of life (Sheiham, 2005). The World Health Organization (WHO) campaign “oral health for a healthier life” highlights the importance of oral cavity as a gateway to the body, reflecting systemic health (Bopp, 2001). Further support for this premise can be found in the literature with several papers indicating the benefits of improving oral hygiene in terms of systemic health one example of this is type 2 diabetes where a bidirectional relationship is proposed (Bascones‐Martinez, Munoz‐Corcuera, & Bascones‐Ilundain, 2015).

As periodontitis progresses to advanced disease it has a major impact on the patient including issues with masticatory function resulting in dietary changes, esthetic concerns, speech difficulties, and self‐esteem issues which may result in social exclusion (Ferreira, Dias‐Pereira, Branco‐de‐Almeida, Martins, & Paiva, 2017; Tonetti, Jepsen, Jin, & Otomo‐Corgel, 2017). However, such consequences could easily be prevented by early intervention to improve oral hygiene (Han & Park, 2017; Lertpimonchai, Rattanasiri, Arj‐Ong Vallibhakara, Attia, & Thakkinstian, 2017); the uptake of appropriate oral hygiene practices is related to the degree of awareness and knowledge the subject has about the oral health (OH) care and periodontal diseases (PD). The adoption of high levels of oral hygiene in industrialized countries has been associated with a reduced prevalence of periodontitis (Kandelman, Arpin, Baez, Baehni, & Petersen, 2012). This was the result of the introduction of intensive educational and preventive oral‐care programs. This is not the case in developing countries, which are lacking in these types of program (Kandelman et al., 2012). Level of awareness of appropriate OH practices and levels of knowledge about PD seem to be affected by a range of factors including gender, age, profession, educational level, systemic conditions, and smoking (Ehizele, Chiwuzie, & Ofili, 2011; Nyorobi, Carneiro, & Kabulwa, 2018; Oberoi, Mohanty, Mahajan, & Oberoi, 2014; Peltzer & Pengpid, 2014; Umeizudike, Onajole, & Ayanbadej, 2015).

Based on the deficiency in the educational programs, the authors hypothesized that awareness and knowledge about OH and PD in Iraqi community are low, and that this would affect the long‐term OH of the population. Thus, this online‐based study aimed to evaluate OH and PD awareness, in the Iraqi population, taking into account a number of demographic variables.

## 2 METHODS

### 2.1 Study design

This study utilized a questionnaire‐based online survey for surveying OH and PD awareness among Iraqi individuals. The survey was open for completion for two weeks. The questionnaire was built using a Google platform and the subsequent link was distributed via social media (Facebook and Telegram) with participants completing the form anonymously. The distribution was performed through sponsored ads and mailing to members of certain Iraqi Facebook groups which were assigned using simple randomization‐lottery method to minimize bias in selection. The issue with participants making more than one entry was excluded by using an IP‐protection protocol. This study was approved by a local ethics committee in the Department of Periodontics, College of Dentistry, University of Baghdad in accordance with Helsinki declaration for human researches. A consent statement was included at the beginning of the questionnaire and agreement was made prior to participation. This study followed the guidelines recommended by STROBE statement for observational studies.

### 2.2 Study sample

The sample was stratified according to the age, the study attempted to include Iraqi subjects over 14 years of age. Iraqi population data, according to the official record, indicated that within this age group there were 24,986,590 subjects (IndexMundi, 2020).

In order to ensure the proposed study had sufficient participants a sample size calculation was performed:

Sample Size = (Distribution of 50%)/([Margin of Error%/Confidence Level Score]^2^ ).

Confidence level = 1.96 (for confidence level of 95%), margin of error = 0.05.

True Sample = (Sample Size × Population)/(Sample Size + Population − 1).

The calculated sample was equal to 385 subjects, which was rounded up to 400, which was then multiplied by 4 to allow 25% response rate (Teoh, Marino, Stewart, & McCullough, 2019); thereby, the final targeted number of respondents was 1,600.

### 2.3 Design of the questionnaire and score calculation

**FIGURE 1:**
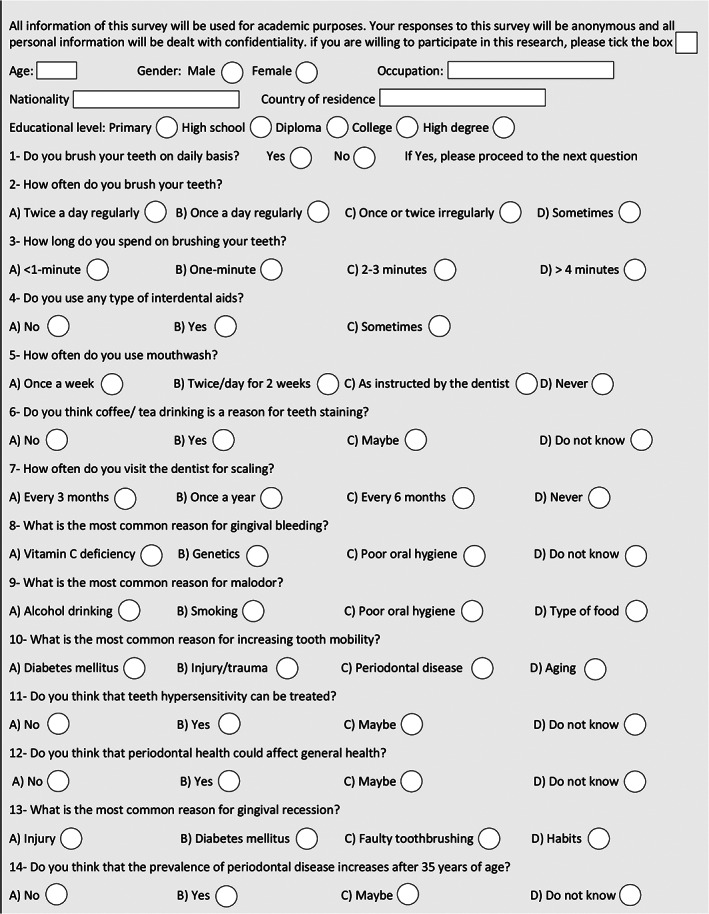
Oral health and periodontal disease awareness questionnaire

The questionnaire has been validated in a previously published research (Dayakar, Kumar, Pai, Shivananda, & Rekha, 2016). It was adapted and then translated into Arabic language for use in the current study. The questionnaire included a demographic data section, which included age, gender, smoking, employment status, educational level, and nationality. Evaluation of awareness was conducted using two main sections; the first one (from question #1 to #7) was dedicated for evaluating OH awareness whereas the second part (from question #8 to #14) was designed to evaluate PD awareness (Figure 1). The questionnaire form was submitted online and distributed via social media for 2 weeks. Each response was marked with “1” for a positive answer and “0” for the other answers. Once each individual response was marked, answers were summed to give an overall score for each of the two sections of the questionnaire; the means were then calculated in order to determine OH and PD awareness. Frequency of the positive answers per question was used to determine the association with different demographic variables. The level of awareness for each section was categorized according to the number of the positive answers per respondent into low (0–3), moderate (4–5), and high (6–7).

The inclusion criteria were Iraqi individuals, aged 14 years and above, having at least primary education and capable of reading and responding to the questionnaire. Any response from non‐Iraqi nationality or Iraqis living in other countries, from a dentist, incomplete, lacking agreement to consent statement was excluded from the final analysis. The exclusion was based on the age, nationality, country of residence, and occupation as reported by the individual.

## 3 STATISTICAL ANALYSIS

Both descriptive and inferential statistics were used for analyzing the data. Comparisons of OH and PD awareness for all participants were determined using Mann–Whitney *U* test. For answers in each awareness section, χ^2^ was used to compare percent of correct response in relation to different demographic variables and levels of knowledge. Association of these variables with correct answers was determined by calculating odds ratio (OR) at 95% confidence interval (CI). All analyzes were performed by using SPSS software (Version 21, IBM, USA). Differences were considered significant when *p* < .05.

## 4 RESULTS

**FIGURE 2:**
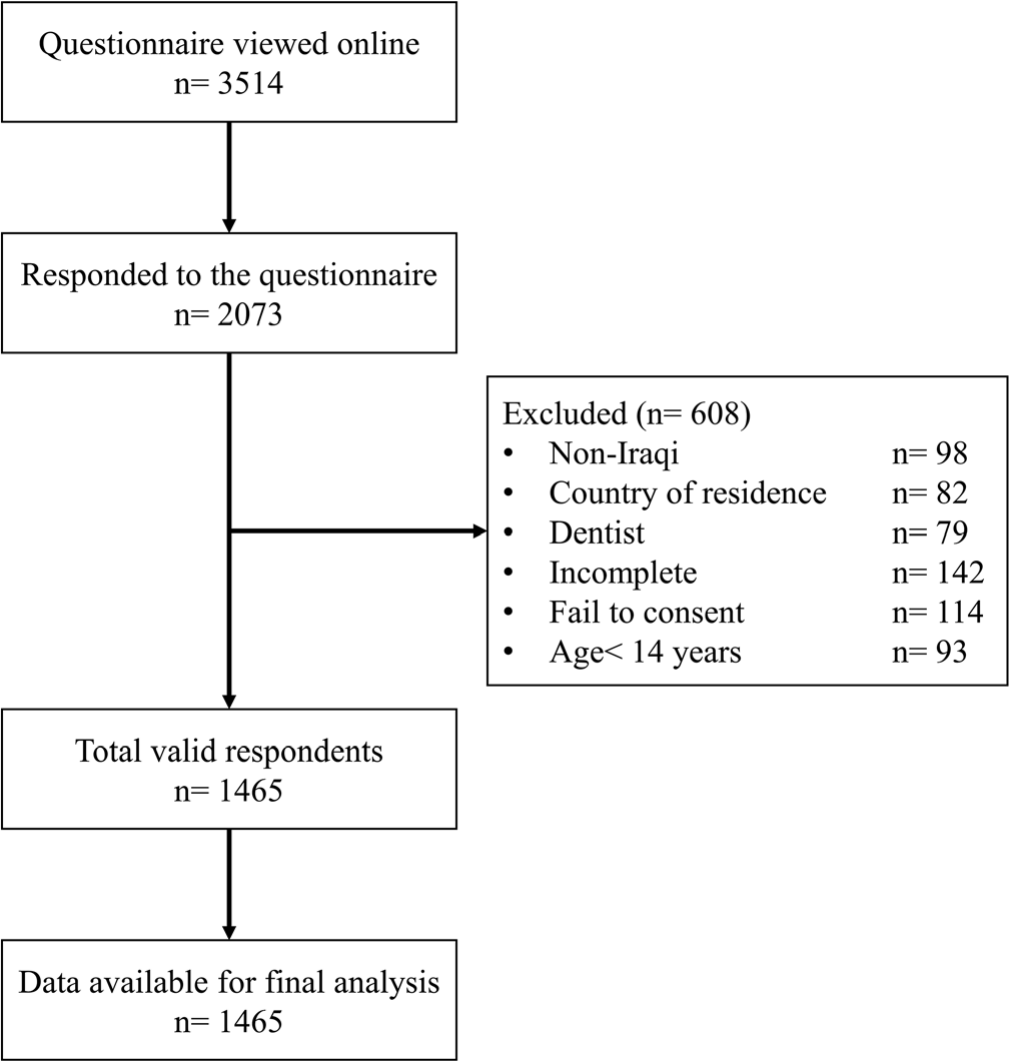
Flow chart of the study

A total of 3,514 viewed the questionnaire online and 2073 had anonymously responded to it, 59% response rate of those who viewed the questionnaire online, of whom 1,465 met the criteria for inclusion and were considered in the final analysis (Figure 2). This number of the respondents was equal to 91.6% of the calculated sample size; thus, it was considered satisfactorily representative for the targeted population. The demographic data of the respondents was summarized in Table 1.

**TABLE 1:** Demographic data of the respondents

Age range (years)	14–72
Mean age ± SD (years)	27.7 ± 8.7
Number of online viewers	3514 (100)^a^
Total respondents	2073 (59)^a^
Total valid respondents	1465 (42)^a^
Gender	
Male	636 (43.4)^a^
Female	829 (56.6)^a^
Smoking	
Yes	241 (16.5)^a^
No	1,224 (83.5)^a^
Employment status	
Not employed	891 (60.8)^a^
Employed	574 (39.2)^a^
Educational level	
Primary	53 (3.6)^a^
High school	234 (16)^a^
Diploma	144 (9.8)^a^
College	814 (55.6)^a^
High degree	220 (15)^a^
Age groups (years)	
14–19	149 (10.2)^a^
20–24	501 (34.2)^a^
25–44	726 (49.6)^a^
>45	89 (6.1)^a^

^a^ Frequency (percentage).

**FIGURE 3:**
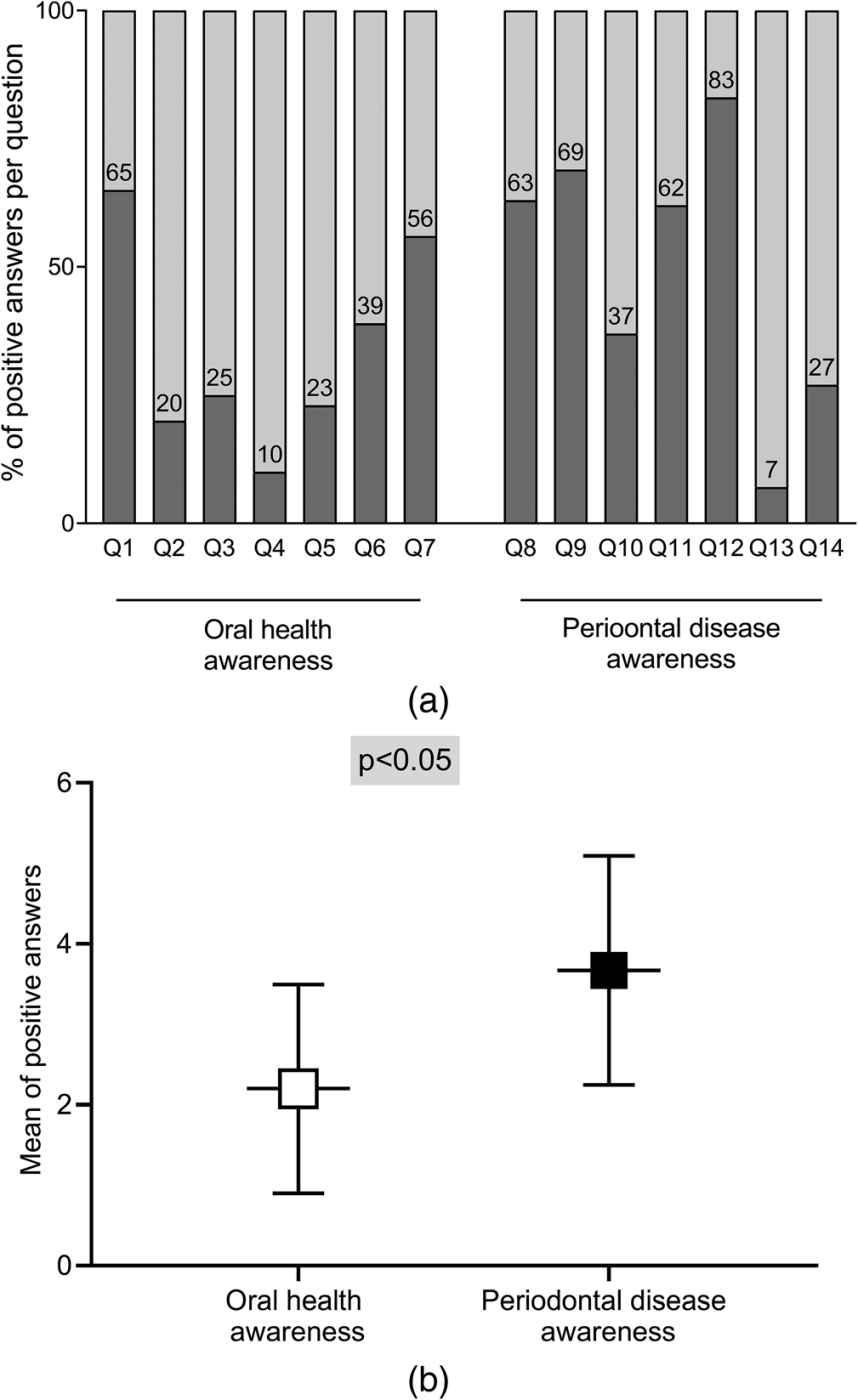
Analysis of responses to oral health awareness questions showed that the highest response rate was associated with toothbrushing practice in which 65% positively answered about brushing, while the lowest response (10%) was associated with the use of interdental aids. For periodontal diseases awareness, about 83% of the respondents showed that they were aware about the effect of periodontal health on the general health; however, only 7% were able to determine the common cause for gingival recession (a). Comparing the mean of answers for both sections of the questionnaire showed that the population had significantly higher periodontal diseases awareness than oral health awareness (b)

The majority of respondents correctly answered the question about the potential adverse effect of PD on the general health. While, the lowest correct response percentage was about the most common reason for gingival recession (Figure 3a). In general, respondents showed significantly higher levels of awareness about PD (median = 4, mean ± SD = 3.66 ± 1.42) than OH awareness (median = 2, mean ± SD = 2.19 ± 1.29) (Figure 3b).

Analysis of answers for OH awareness questions according to the demographic variables is shown in Table 2. In general, non‐smoker, ≥45 years, female, and respondents who holding high educational degrees brushed their teeth twice a day on regular basis more than their counterparts. No significant difference in the reported time taken for tooth brushing with exception for age groups in which respondents ≥45 years were the highest. The only significant difference for the use of interdental aids was observed according to educational level as the high degree holders were the highest and age groups as the oldest subjects being the highest. The responses for using mouthwashes, results showed significant difference between female versus male, and employed versus non‐employed. Results according to educational level, and age groups showed a similar pattern to the responses for the previous question. Answers about the potential staining effect of tea and coffee on teeth showed only significant differences in relation to gender and educational level. Frequency of visiting dentists once every 6 months showed significant difference between employed and non‐employed, and age groups where the age group 25–44 years was the reported to show most frequent attendance.

**TABLE 2:** Oral health awareness according to the demographic variables (frequency of positive responses, percentage [%])

	Q1	Q2	Q3	Q4	Q5	Q6	Q7	Odds ratio (OR)**	95% CI
Gender									
Male	334, 52.5%	100, 15.7%	173, 27.2%	71, 11.1%	128, 20.1%	391, 61.5%	36, 5.7%	1	
Female	616, 74.3%	192, 23.2%	197, 23.8%	82, 9.9%	216 26.1%	625, 75.4%	60, 7.2%	1.360	1.249–2.963
	<0.001*	<0.001*	0.133*	0.430*	0.008*	<0.001*	0.227*		
Smoking									
Yes	115, 47.7%	269, 21.9%	65, 26.9%	28, 11.6%	49, 20.3%	163, 67.6%	15, 6.2%	1	
No	835, 68.2%	23, 9.5%	305, 24.9%	125, 10.2%	295, 24.1%	853, 69.7%	81, 6.6%	1.277	1.137–1.436
	<0.001*	<0.001*	0.503*	0.514*	0.207*	0.527*	0.821*		
Employment level									
Yes	391, 68.1%	122, 21.3%	140, 24.4%	68, 11.9%	157, 27.4%	395, 68.8%	50, 8.7%	1	
No	559, 62.7%	170, 19.1%	230, 25.8%	85, 9.5%	187, 20.9%	621, 69.7%	46, 5.2%	1.122	1.031–2.444
	0.035*	0.309*	0.540*	0.159*	0.005*	0.721*	0.007*		
Educational level									
Primary	29, 54.7%	8, 15.1%	9, 17%	6, 11.3%	12, 22.6%	34, 64.2%	3, 5.7%	1	
High school	111, 47.4%	31, 13.2%	54, 23.1%	23, 9.8%	35, 15%	146, 62%	10, 4%	0.893	0.694–1.151
Diploma	75, 52.1%	23, 16%	34, 23.6%	10, 6.9%	34, 23.6%	89, 61.8%	9, 6.3%	0.998	0.767–1.307
College	557, 68.4%	161, 19.8%	215, 26.4%	73, 9%	180, 22.1%	592, 72.7%	53, 6.5%	1.266	1.002–1.603
High degree	178, 80.9%	69, 31.4%	58, 26.4%	41, 18.6%	83, 37.7%	155, 70.5%	21, 9.5%	1.730	1.345–2.214
	<0.001*	<0.001*	0.487*	<0.001*	<0.001*	0.006*	0.258*		
Age groups (years)									
14–19	93, 62.4%	32, 21.5%	40, 26.8%	15, 10.1%	17, 11.4%	96, 64.4%	12, 8.1%	1	
20–24	305, 60.9%	83, 16.6%	141, 28.1%	35, 7%	94, 18.8%	335, 66.9%	20, 4%	0.983	0.845–1.146
25–44	475, 65.4%	146, 20.1%	160, 22%	77, 10.6%	193, 26.6%	521, 71.8%	61, 8.4%	1.146	0.990–1.327
≥45	77, 86.5%	31, 34.8%	29, 32.6%	26, 29.2%	40, 44.9%	64, 71.9%	3, 3.4%	1.851	1.505–2.276
	<0.001*	0.001*	0.030*	<0.001*	<0.001*	0.144*	0.010*		

*Note*: *Significance level at *p* < .05 by χ2 test. **OR at 95% CI by Baptista‐Pike test. Q1: Do you brush your teeth on daily basis?; Q2: How often do you brush your teeth?; Q3: How long do you spend on brushing your teeth?; Q4: Do you use any type of interdental aids?; Q5: How often do you use mouthwash?; Q6: Do you think coffee/ tea drinking is a reason for teeth staining?; Q7: How often do you visit the dentist for scaling?.

By considering the demographic variables in the analysis, all groups showed no statistically significant difference in responding to the main reasons for gingival bleeding, and tooth mobility (except for specific age groups). In contrast, all groups showed significant differences in their responses relating to the main cause of malodor (except for specific age groups). The responses to the ability of treating tooth hypersensitivity indicated significant difference according to gender and education level. Potential effect of periodontal health on the general health was significantly agreed by non‐smokers than smokers, by college degree and higher degree holders relative to other educational levels, in addition to age groups. The non‐smoker and non‐employed respondents were significantly more aware about the involvement of PD in gingival recession. Responses of increasing prevalence of PD with aging showed significant difference according to educational level and age (Table 3).

**TABLE 3:** Periodontal diseases awareness according to the demographic variables (frequency of positive responses, percentage [%])

	Q8	Q9	Q10	Q11	Q12	Q13	Q14	Odds ratio (OR)**	95% CI
Gender									
Male	244, 38.4%	367, 57.7%	244, 38.4%	373, 58.7%	522, 82.1%	349, 54.9%	174, 27.4%	1	
Female	322, 38.8%	560, 67.6%	302, 36.4%	530, 63.9%	696, 83.9%	472, 56.9%	220, 26.5%	1.101	1.018–1.190
	0.853*	<0.001*	0.448*	0.039*	0.341*	0.431*	0.726*		
Smoking									
Yes	99, 41.4%	126, 52.3%	95, 39.4%	150, 62.2%	179, 74.3%	118, 48.9%	79, 32.8%	1	
No	467, 38.2%	801, 65.4%	451, 36.9%	753, 61.5%	1,039, 84.9%	703, 57.4%	315, 25.7%	1.115	1.004–1.238
	0.394*	<0.001*	0.450*	0.833*	<0.001*	0.015*	0.024*		
Employment level									
Yes	218, 37.9%	341, 59.4%	197, 34.3%	346, 60.1%	490, 85.4%	299, 52.1%	168, 29.3%	1	
No	348, 39.5%	586, 65.8%	349, 39.2%	557, 62.5%	728, 81.7%	522, 58.6%	226, 25.4%	0.925	0.428–1.002
	0.572*	0.014*	0.061*	0.390*	0.068*	0.014*	0.100*		
Educational level									
Primary	17, 32.1%	22, 41.5%	20, 37.7%	26, 49.1%	41, 77.4%	27, 50.9%	19, 35.8%	1	
High school	95, 41.3%	144, 62.2%	96, 41.7%	136, 59.1%	178, 77.4%	128, 55.7%	52, 8.3%	1.186	0.947–1.489
Diploma	45, 31.3%	80, 55.6%	63, 43.8%	81, 56.3%	111, 77.1%	77, 53.5%	32, 13.2%	1.090	0.861–1.384
College	322, 39.6%	537, 66%	299, 36.7%	511, 62.8%	694, 85.3%	472, 58%	215, 2.3%	1.333	1.080–1.644
High degree	87, 39.5%	144, 65.5%	68, 30.9%	149, 67.7%	194, 88.2%	117, 53.2%	78, 8.6%	1.370	0.546–1.719
	0.256*	0.001*	0.095*	0.034*	<0.001*	0.542*	0.011*		
Age groups (years)									
14–19	59, 39.6%	95, 63.8%	59, 39.6%	82, 55%	123, 82.6%	95, 63.8%	21, 14.1%	1	
20–24	201, 40.1%	332, 66.3%	213, 42.5%	311, 62.1%	399, 79.6%	280, 55.9%	116, 23.2%	1.067	0.930–1.225
25–44	277, 38.2%	453, 62.4%	253, 34.8%	447, 61.6%	612, 84.3%	404, 55.6%	220, 30.3%	1.052	0.460–1.202
≥45	29, 32.6%	47, 52.8%	21, 23.6%	63, 70.8%	84, 94.4%	42, 47.2%	37, 41.6%	1.026	0.840–1.252
	0.579*	0.094*	0.002*	0.115*	0.004*	0.090*	<0.001*		

*Note*: *Significance level at *p* < .05 by χ^2^ test. **OR at 95% CI by Baptista‐Pike test. Q8: What is the most common reason for gingival bleeding?; Q9: What is the most common reason for malodor?; Q10: What is the most common reason for increasing tooth mobility?; Q11: Do you think that teeth hypersensitivity can be treated?; Q12: Do you think that periodontal health could affect general health?; Q13: What is the most common reason for gingival recession?; Q14: Do you think that the prevalence of periodontal diseases increases after 35 years of age?.

In the logistic regression analysis of different factors that were independently associated, (at 95% CI), with OH awareness included; gender (OR 1.360), smoking status (OR 1.277), employment (OR 1.122), educational level (College OR 1.266 and high degree OR 1.730), and age ≥ 45 (OR 1.851) (Table 2). PD awareness was associated with gender (OR 1.101), smoking status (OR 1.115), and educational level (college OR 1.333, high degree OR 1.370); however, no association was seen with employment status and age groups (Table 3).

Some of the respondents in this study exhibited low levels of OH knowledge; significant differences were observed between gender, educational level, and age groups (*p* < .05). Interestingly, they also had low to moderate level of PD knowledge (Table 4).

**TABLE 4:** Knowledge level of the study population according to the demographic variables

	Oral health knowledge			Periodontal diseases knowledge		
	Low	Moderate	High	Low	Moderate	High
Gender						
Male	87.3%	12.4%	0.3%	47.5%	43.9%	8.6%
Female	81.5%	17%	1.4%	42.1%	46.4%	11.6%
		0.004*			0.054*	
Smoking						
Yes	89.2%	10%	0.8%	50.2%	42.7%	7.1%
No	83%	16%	1%	43.3%	45.8%	10.9%
		0.053*			0.063*	
Employment						
No	85.9%	13.2%	0.9%	42.8%	46.5%	10.7%
Yes	81.2%	17.8%	1%	47%	43.4%	9.6%
		0.056*			0.265*	
Educational level						
Primary	88.7%	11.3%	0%	54.7%	41.5%	3.8%
High school	93.2%	6.4%	0.4%	48.7%	41%	10.3%
Diploma	87.5%	11.1%	1.4%	52.8%	41%	6.3%
College	84.3%	14.7%	1%	42.4%	46.4%	11.2%
High degree	70%	28.6%	1.4%	39.5%	49.1%	11.4%
		<0.001*			0.072*	
Age groups (years)						
14–19	87.2%	10.7%	2%	47%	45.6%	7.4%
20–24	88.4%	11.2%	0.4%	41.9%	47.5%	10.6%
25–44	82.9%	16.1%	1%	45.6%	43.7%	10.7%
≥45	62.9%	34.8%	2.2%	44.9%	44.9%	10.1%
		<0.001*			0.739*	

*Note*: *Significance level at *p* < .05 by χ^2^ test.

## 5 DISCUSSION

The current study used online‐based platform to distribute the questionnaire via social media websites including Facebook and Telegram. The use of social media is found widely in the population and allows effective and rapid sharing of information (Boyd & Ellison, 2007). Worldwide, approximately 2.7 billion subscribers use the social media, with about 1.52 billion daily active users of Facebook alone (Petosic, Sunde, Beeckman, Flaatten, & Wøien, 2019). A questionnaire‐based survey, involving physicians and nurses to evaluate the use of social media showed 64% response rate, has suggested that this media can be utilized as alternative platform for communications (Petosic et al., 2019). The current study showed a response rate close to that reported in literature reaching up to 59%. In other words, this study's response rate is satisfactorily representing the targeted population. However, online‐based questionnaire has a limitation in which it cannot be accessed by certain members of the population most notably those who do not have internet access or IT illiterate, and potentially elderly subjects. This agrees with the current results, which showed that all respondents have certain degree of educational level and a relatively young mean of age (27.7 years) of the participants. The responses were selected from individuals not having a previous academic dental education; however, they showed moderate level of awareness and knowledge about PD which were significantly associated with gender and educational level. These results are consistent with a previous study conducted on non‐medical professionals in Nigeria whom PD knowledge was relatively high but not oral hygiene practice (Umeizudike et al., 2015).

OH knowledge is an integral attitude to preserve a healthy oral cavity. Studies have indicated that subjects acquire this knowledge from different sources such as media (e.g., newspapers, radio, and television), self‐experience, school, parents, and even from friends or relatives (d'Almeida, Kagami, Maki, & Takaesu, 1997; Gholami, Pakdaman, Montazeri, Jafari, & Virtanen, 2014; Martensson, Soderfeldt, Halling, & Renvert, 2004; Miller, Lee, DeWalt, & Vann Jr, 2010). Unfortunately, most of these sources provide unreliable information and only few individuals follow the printed or verbal instructions given by dentists (Masood Mirza, Khan, Ali, & Chaudhry, 2007). Results from meta‐analysis and systematic reviews recommended tooth brushing twice a day, in the morning and at bedtime, together with the use of appropriate interdental aids and regular dental attendance as suitable approaches to prevent PD (Lertpimonchai et al., 2017). Almost half of the sample positively responded to the frequency of tooth brushing but not visiting the dentist for regular examinations, which showed less than 10% response rate. A previous survey conducted in Kuwait showed that about two‐thirds of the participants brush twice a day; yet, majority of them had not visited their dentist for regular examinations in the last 6 months (Al‐Shammari, Al‐Ansari, Al‐Khabbaz, Dashti, & Honkala, 2007). Commitment to regular twice a day tooth brushing showed significant positive association with increasing age, which is inconsistent with a previous study that showed the opposite association with geriatric patients (Kulshreshta, 2017). This could be related to differences in sample size or the method of collecting the information in which the online survey used in this study, may attract individuals with higher educational level as compared to the use of a paper‐based questionnaire. Further, responses of older individuals in this study to questions regarding the use of interdental aid and mouthwash showed higher rate as compared to younger individuals, which suggests enhanced awareness of OH awareness as subjects get older. This was supported by data generated in this study, which showed significantly higher levels of knowledge among individuals aged ≥45 years. This may be explained by cumulative knowledge through lifetime acquired from previous dental experiences potentially encouraging older individuals to preserve their remaining teeth. According to gender, females showed significantly higher OH awareness than male counterparts especially of daily tooth‐care practices such as frequency of brushing, use of mouthwashes, and awareness about causes of teeth staining that may compromise their esthetics. These findings are in agreement with studies conducted in Spain and USA that have utilized comprehensive measure of oral hygiene knowledge in which females exhibited higher OH knowledge levels than males (Macek et al., 2010; Macek et al., 2017; Márquez‐Arrico, Almerich‐Silla, & Montiel‐Company, 2019). Mouthwashes, which can be prescribed as adjunctive treatment when tooth brushing is inadequately in controlling the dental plaque. Majority of the respondents reported that they were using mouthwashes without referring to their dentists together with significant differences in association with gender, employment status, education, and age. Similar to findings from previous study, a low percentage of respondents used mouthwash according to the instructions provided by their dentist (Al‐Shammari et al., 2007). This highlighted a deficiency in the awareness of the indications for mouthwash use. Another area that showed low levels of knowledge was the use of interdental aids; a frequent finding in other international studies (Al‐Shammari et al., 2007; Ehizele et al., 2011; Umeizudike et al., 2015).

A further finding from this study was that employed individuals showed higher OH awareness than unemployed. Indeed, the stress of not having a job and a regular income can negatively impact on quality of the life which is in turn can be reflected in neglecting oral hygiene, this can further be exacerbated by the cost implications in visiting a dentist. This lack of engagement in dental care is exacerbated by the absence of a healthcare insurance system supporting basic dental treatment. However, the levels of OH and PD knowledge of the respondents showed no significant difference between employed and unemployed individuals. Similar findings were reported by another study conducted in UAE, which also showed no effect of employment status on the level of OH knowledge scores (Abu‐Gharbieh, Saddik, El‐Faramawi, Hamidi, & Basheti, 2019). In addition to the profession, formal education was found to be positively correlated with knowledge about OH and PD, this has been highlighted in other published studies (Azodo & Omili, 2014; Nyorobi et al., 2018; Zavras, Vrahopoulos, Souliotis, Silvestros, & Vrotsos, 2002). Our findings suggested the presence of association between educational level and level of oral hygiene and PD knowledge, which is reflected by a significantly higher awareness, the same pattern, was reported by previous surveys (Macek et al., 2010; Márquez‐Arrico et al., 2019; Nyorobi et al., 2018). Individuals with unfavorable habits such as smoking, that is known to cause deterioration of OH and systemic health, will potentially show lower concern about their levels of oral hygiene, this can be seen in the findings of the current study and has been clearly shown in a number of other studies(Abu‐Gharbieh et al., 2019).

Generally, majority of the responses indicated low levels of oral hygiene knowledge in relation to all demographic variables included in this survey, which is similar to the previous published results (Macek et al., 2010). Interestingly, the knowledge about PD was found to be higher than OH knowledge, with responses almost equally distributed between “Low” and “Moderate” levels. There is no clear explanation for such variation in the knowledge of the two sections of the survey; nevertheless, advanced PD is more tangible by the subjects that may motivate them to seek information about the signs and treatments for these conditions. Another finding can be comprehended from the analysis that oral hygiene behavior was also deficient among the respondents. This was reflected by answers to questions about oral hygiene practices in the questionnaire such as details of tooth brushing, use of interdental aids and mouthwashes.

Limitation to access the internet or lack of the required knowledge to use it suggest using other methods of surveys such as paper‐based or interviews to reach other members of Iraqi community who were not able to be engaged in the current online‐surveys.

## 6 CONCLUSIONS

Despite limitations, this study revealed inappropriate levels of OH and PD awareness and knowledge in the Iraqi population and provided the baseline data necessary for the development of Governmental educational programs and health awareness campaigns, which are highly suggested particularly focusing on the primary and high schools, in an attempt to improve the levels of awareness.

## CONFLICT OF INTEREST

The authors declare no potential conflict of interest.

## AUTHOR CONTRIBUTIONS

Hayder R. Abdulbaqi and Ali A. Abdulkareem were responsible for research concept and design. Muhanad L. Alshami collected the data, Ali A. Abdulkareem and Hayder R. Abdulbaqi analyzed the results. Mike R. Milward was responsible for proofreading the final version of the manuscript and critical revision of the article. All authors contributed in writing and approving the final draft of the manuscript.
